# Active Transport of Phosphorylated Carbohydrates Promotes Intestinal Colonization and Transmission of a Bacterial Pathogen

**DOI:** 10.1371/journal.ppat.1005107

**Published:** 2015-08-21

**Authors:** Brandon Sit, Shauna M. Crowley, Kirandeep Bhullar, Christine Chieh-Lin Lai, Calvin Tang, Yogesh Hooda, Charles Calmettes, Husain Khambati, Caixia Ma, John H. Brumell, Anthony B. Schryvers, Bruce A. Vallance, Trevor F. Moraes

**Affiliations:** 1 Department of Biochemistry, University of Toronto, Toronto, Ontario, Canada; 2 Department of Pediatrics and the Child and Family Research Institute, University of British Columbia, Vancouver, British Columbia, Canada; 3 Department of Microbiology and Infectious Diseases, University of Calgary, Calgary, Alberta, Canada; 4 Department of Biochemistry and Molecular Biology, University of Calgary, Calgary, Alberta, Canada; 5 Department of Molecular Genetics and Institute of Medical Science, University of Toronto, Ontario, Canada; 6 Program in Cell Biology, Hospital for Sick Children, Toronto, Ontario, Canada; 7 SickKids Inflammatory Bowel Disease Centre, Toronto, Ontario, Canada; Northwestern University Feinberg School of Medicine, UNITED STATES

## Abstract

Efficient acquisition of extracellular nutrients is essential for bacterial pathogenesis, however the identities and mechanisms for transport of many of these substrates remain unclear. Here, we investigate the predicted iron-binding transporter AfuABC and its role in bacterial pathogenesis *in vivo*. By crystallographic, biophysical and *in vivo* approaches, we show that AfuABC is in fact a cyclic hexose/heptose-phosphate transporter with high selectivity and specificity for a set of ubiquitous metabolites (glucose-6-phosphate, fructose-6-phosphate and sedoheptulose-7-phosphate). AfuABC is conserved across a wide range of bacterial genera, including the enteric pathogens EHEC O157:H7 and its murine-specific relative *Citrobacter rodentium*, where it lies adjacent to genes implicated in sugar sensing and acquisition. *C*. *rodentium ΔafuA* was significantly impaired in an *in vivo* murine competitive assay as well as its ability to transmit infection from an afflicted to a naïve murine host. Sugar-phosphates were present in normal and infected intestinal mucus and stool samples, indicating that these metabolites are available within the intestinal lumen for enteric bacteria to import during infection. Our study shows that AfuABC-dependent uptake of sugar-phosphates plays a critical role during enteric bacterial infection and uncovers previously unrecognized roles for these metabolites as important contributors to successful pathogenesis.

## Author Summary

Essentially all Gram-negative pathogens are reliant on specific transport machineries termed binding protein-dependent transporters (BPDTs) to transport solutes such as amino acids, sugars and metal ions across their membranes. In this study we investigated AfuABC, a predicted iron-transporting BPDT found in many bacterial pathogens. We show by structural and functional approaches that AfuABC is not an iron transporter. Instead, AfuABC is a trio of proteins that bind and transport sugar-phosphates such as glucose-6-phosphate (G6P). In doing so, we present the first structural solution of a G6P-specific transport protein and add to the few known unique machineries for sugar-phosphate uptake by bacteria. Furthermore, we show that AfuABC is required by the intestinal pathogen *C*. *rodentium* to effectively transmit between mice and re-establish infection, leading us to propose that the transport of sugar-phosphates is an important part of general bacterial pathogenesis.

## Introduction

Competition between host cells and pathogenic bacteria for diverse substrates ranging from metals to vitamins can often influence the outcome of infection. As a result, pathogens have developed a variety of nutrient uptake mechanisms to increase their competitiveness and hence their ability to colonize and successfully infect their hosts [1]. One such example of these nutrient uptake mechanisms are the binding-protein dependent transporters (BPDTs). BPDTs are ubiquitous in bacteria and rely on the presence of a soluble ligand binding protein that directs a specific substrate to an integral membrane permease/ATPase complex. The ensuing tripartite complex then couples the hydrolysis of cytosolic ATP to the influx of the ligand into the cytosol for downstream uses [2]. BPDTs target diverse ligands such as metal ions, amino acids and sugars and are recognized as both virulence determinants and therapeutic targets in many bacterial infections [2].

In this study, we investigate AfuABC (**A**ctinobacillus **f**erric **u**ptake ABC), a BPDT originally identified as an iron-specific transporter in *Actinobacillus pleuropneumoniae*, a Gram-negative upper respiratory tract pathogen [3]. The locus *afuABC* encodes 3 polypeptides corresponding to a conventional BPDT–AfuA (a periplasmic binding protein or PBP), AfuB (an inner membrane permease), and AfuC (a cytosolic ATPase). Although AfuABC is annotated as an Fe^3+^-binding transporter, its ligand has been the subject of controversy [4–6]. Initially of interest given the central role of iron piracy to the success of bacterial pathogens, AfuABC has not been investigated on a functional level and the role of currently annotated AfuABC homologues in virulence of other bacterial species has not been characterized. Since several studies have identified *afuABC* as transcriptionally upregulated during bacterial infection [7–9], we investigated AfuABC on a structural and functional basis to definitively identify its ligand and further illuminate its role in pathogenesis. We found that AfuABC is not an iron transporter as previously described and instead that this BPDT is specific for phosphorylated sugars. AfuABC contributed to virulence and was key to transmission success in a mouse model of *C*. *rodentium* infection, highlighting its role in nutrient uptake during bacterial pathogenesis.

## Results

### AfuA is a sugar-phosphate specific periplasmic binding protein

**Fig 1:**
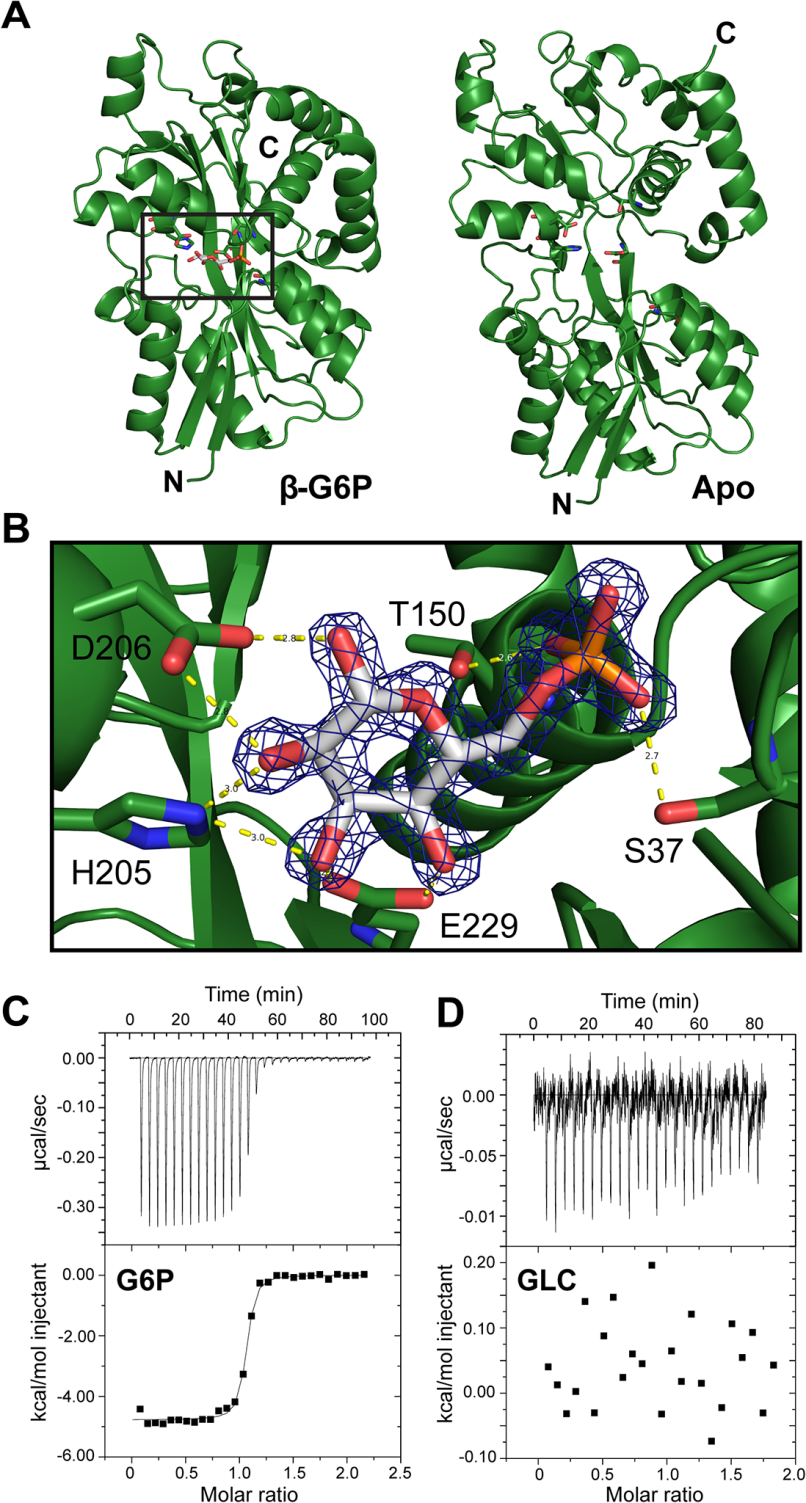
AfuA is a sugar-phosphate specific periplasmic binding protein. (A) Ribbon diagrams of *A*.*pleuropneumoniae* AfuA complexed with either no ligand (right) or β-G6P (left). (B) Cleft region of AfuA depicting 2F_0_-F_c_ map contoured at 2σ around β-G6P. Residues used for mutagenic studies are indicated with text labels. Predicted hydrogen bonds are drawn as yellow dashed lines with distances indicated in angstroms (Å). (C) Representative ITC curve for a positive AfuA-ligand interaction. The curve shown is from a titration of *A*.*pleuropneumoniae* AfuA with a 10:1 ratio of G6P. (D) Representative ITC curve illustrating lack of an AfuA-ligand interaction. The curve shown is from a titration of *A*.*pleuropneumoniae* AfuA with a 10:1 ratio of glucose.

To structurally characterize the ligand of AfuA, a 1.6Å crystal structure of ligand-bound *A*. *pleuropneumoniae* AfuA purified from *E*. *coli* was solved by use of sulfur single wavelength anomalous diffraction. AfuA is a class II/cluster D periplasmic binding protein with two globular α/β domains linked by a dual β-stranded hinge [10] (S1 Table and Fig 1A). In the binding cleft, we unexpectedly observed positive electron density and identified glucose-6-phosphate (β-G6P) as the bound ligand with a small (~5%) portion of density at carbon 2 corresponding to mannose-6-phosphate (β-M6P) (Fig 1B). In the binding cleft, His205, Asp206 and Glu229 interact multivalently with the sugar ring while Ser37 and Thr150 form hydrogen bonds with the phosphate moiety of G6P (Fig 1B). Isothermal titration calorimetry (ITC) validated the binding affinity of *A*. *pleuropneumoniae* AfuA for β-G6P at 24 nM with favourable values of enthalpy and entropy change (S2 Table and Fig 1C and 1D). In order to probe the ligand-binding site of AfuA, site-directed mutants were generated for putative binding residues. The mutations T150A and S37A lowered but did not eliminate binding to G6P, whereas mutations to the sugar-binding residues (H205A, D206A and E229A) completely abrogated the interaction (Table 1). This indicates that the hydrogen-bonding capacity of the sugar-coordinating residues is the critical determinant in mediating binding of potential sugar phosphates.

**Table 1:** Binding constants between *A*.*pleuropneumoniae* AfuA and G6P.

Mutant	K_d_ (μM)	Sites (N)
**WT**	0.023 ± 0.004	1.06 ± 0.04
**S37A**	0.9 ± 0.1	1.01 ± 0.02
**S37D**	5.5 ± 0.9	1.04 ± 0.06
**T150A**	3.3 ± 0.2	1.14 ± 0.03
**H205A**	No binding	
**D206A**	No binding	
**E229A**	No binding	

Binding constants were determined by ITC as described in the Materials and Methods. Values shown are the mean result from three independent AfuA purifications ± SEM.

**Fig 2:**
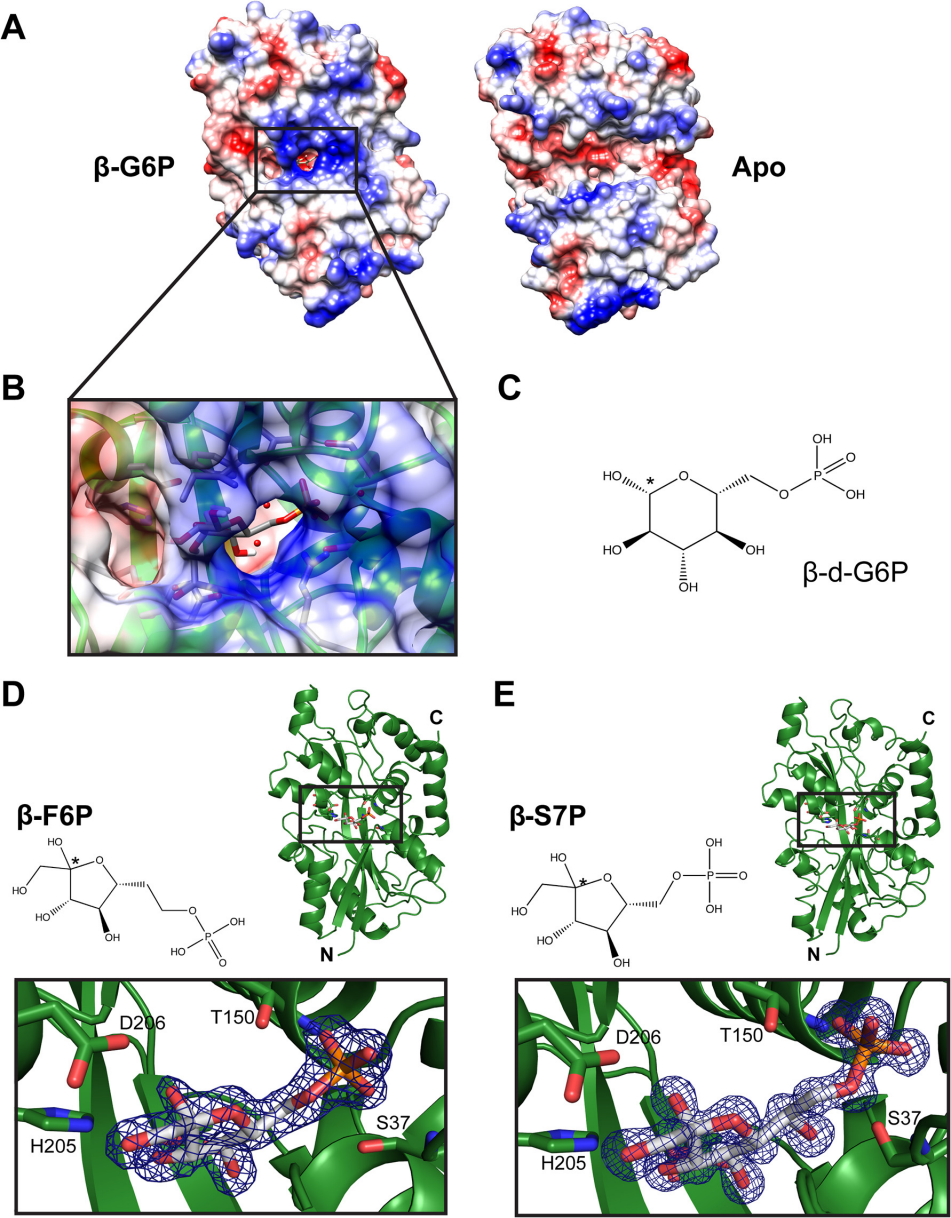
Electrostatic surface mapping of AfuA and additional ligand-bound structures. (A) Electrostatic surface potential of apo (right) and G6P-bound (left) AfuA. Potential values range from -10 (red) to +10 kJ/mol·e (blue). (B) Magnified view of G6P-AfuA binding cleft with electrostatic surface map overlay. Residues proximal to the G6P molecule are shown as sticks. Water molecules are shown as red spheres. (C) Chemical structure of β-G6P. * denotes the anomeric carbon. (D, E) Ribbon diagrams showing *A*.*pleuropneumoniae* AfuA in complex with (D) F6P or (E) S7P. Inset boxes below each ribbon structure show the 2F_0_-F_c_ map contoured at 2σ around each ligand.

To gain insight into the mechanism of ligand capture by this PBP, a structure of apo (open)-AfuA was solved to 1.6Å. The globular domains of apo-AfuA are held at a 29.7° angle relative to the closed form (Fig 1A). The binding pocket of AfuA contains both electropositive and electronegative regions, corresponding to the regions of the pocket that bound the phosphate or sugar moiety of G6P respectively (Fig 2A, 2B and 2C). A subsequent ligand screen by ITC with structurally similar molecules revealed that three other sugar-phosphates including fructose-6-phosphate (F6P), sedoheptulose-7-phosphate (S7P) and M6P had affinities of 8, 57 and 960 nM respectively for AfuA (Table 2 and S1 Fig). F6P binding affinities of the single site AfuA mutants displayed the same trend as for G6P, suggesting a similar binding mechanism between the two ligands (S3 Table). Structures of AfuA bound to F6P (2.0Å) and S7P (1.3Å) were also solved to confirm ligand coordination. Similar to the G6P structure, both F6P and S7P were found in the β-anomer conformation (Fig 2D and 2E). Between the three holo-AfuA structures, strand and amino acid placement and conformation were identical (RMSD < 0.4Å between C_α_ chains). Interestingly, S7P was captured by AfuA in its furan form and not pyran as typically depicted. S7P and its derivatives can exist as furan forms *in vivo* [11], indicating that this ligand structure is biologically relevant. AfuA did not bind other sugar-phosphates such as glucose-1-phosphate and ribose-5-phosphate, demonstrating a high level of selectivity for ligands (Table 2 and S1 Fig). No interaction between AfuA and glucose, fructose or inorganic phosphate could be detected (Table 2 and S1 Fig). These data collectively show that AfuA is a PBP that is highly specific for four sugar-phosphates involved in central metabolism.

**Table 2:** Binding constants between WT *A*.*pleuropneumoniae* AfuA and screened molecules.

Ligand	K_d_ (nM)
**Glucose-6-phosphate**	23 ± 4
**Fructose-6-phosphate**	8 ± 1
**Sedoheptulose-7-phosphate**	57 ± 22
**Mannose-6-phosphate**	960 ± 150
**Glucose**	No binding
**Fructose**	No binding
**Glucose-1-phosphate**	No binding
**Ribose-5-phosphate**	No binding
**Fructose-1,6-bisphosphate**	No binding
**Na**_**2**_ **HPO**_**4**_	No binding

Binding constants were determined by ITC as described in the Materials and Methods. Values shown are the mean result from three independent AfuA purifications ± SEM.

### AfuABC is a *bona fide* sugar phosphate-specific BPDT

**Fig 3:**
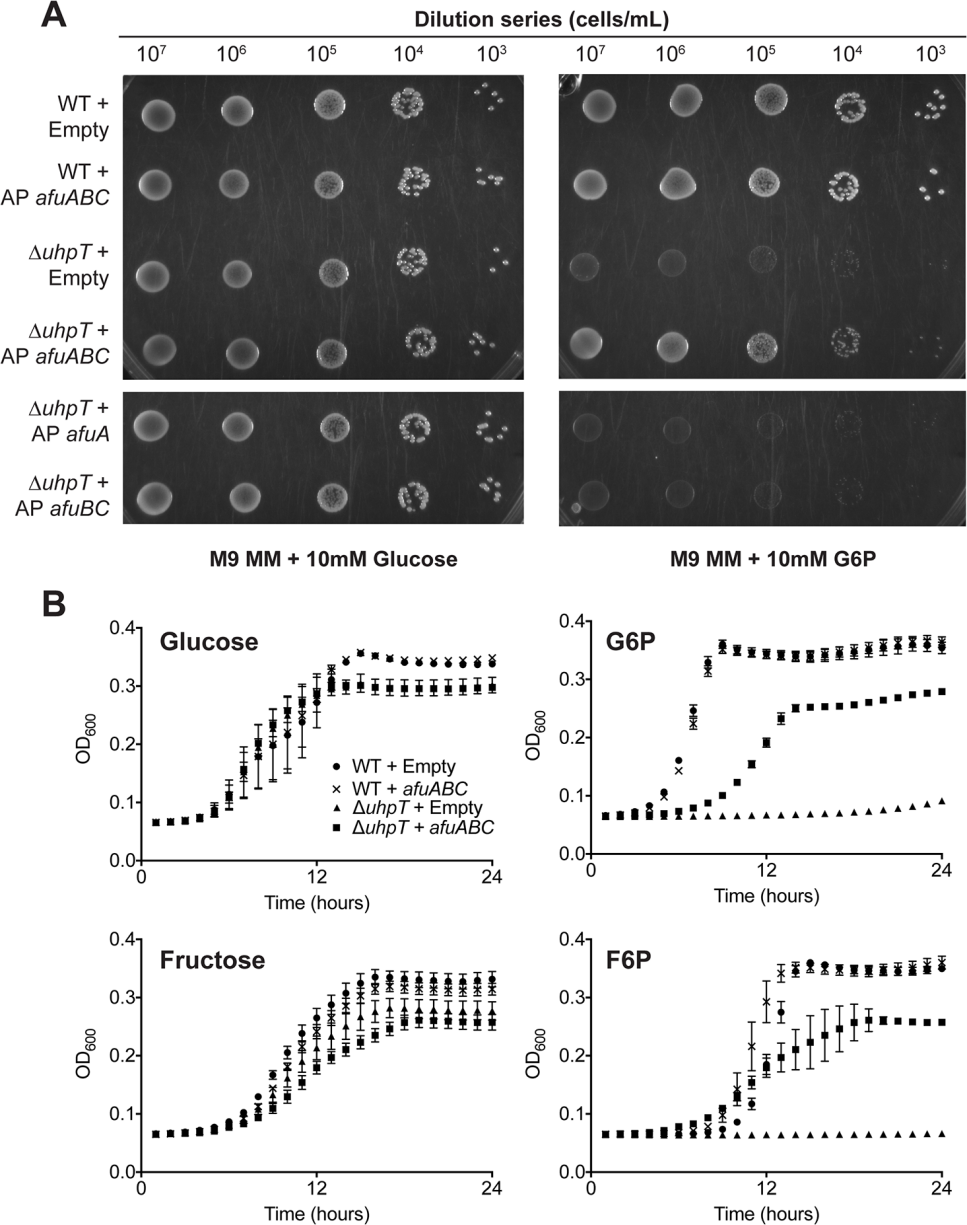
*afuABC* rescues a nutrient-limited *E*.*coli* strain. (A) Solid media complementation of Δ*uhpT* in M9 minimal media (M9 MM) supplemented with 10mM glucose or G6P. 5μL drops were plated of the indicated dilutions and grown at 37°C for 30 hours before imaging. Plates are representative of n = 3 transformations. (B): OD_600_ readings of *E*.*coli* growth over 24 hours at 37°C in M9 minimal medium supplemented with 10mM glucose, G6P, fructose or F6P. Readings were taken every 15 minutes–data shown is parsed to hourly readings for clarity. Curve legend is the same in all panels and is indicated in the first panel. Error bars represent SEM of cell growth from n = 3 transformations.

Since AfuA binds sugar-phosphates, we queried whether AfuABC could recapitulate sugar-phosphate transport in a bacterium that could not transport these substrates. The only other sugar-phosphate uptake system identified thus far in Gram-negative bacteria is the two component system UhpABCT. UhpT is an inner membrane antiporter that couples the influx of hexose phosphates such as G6P and F6P to the efflux of inorganic phosphate [12]. The expression of UhpT is regulated by the transcription factor UhpA, which is activated in response to periplasmic sugar-phosphates through the inner membrane sensor kinase complex UhpB/UhpC [13]. *E*. *coli ΔuhpT* were unable to replicate in medium where G6P or F6P were the only carbon source, confirming UhpT is the only hexose-phosphate specific transporter in BW25113 (Figs 3A, 3B and S2) [14]. When *ΔuhpT* cells were transformed with a vector carrying the *A*. *pleuropneumoniae afuABC* operon, a significant increase in growth rate on M9 + G6P or F6P agar media compared to empty vector-transformed cells was observed (S4 Table and Figs 3A and S2A). This growth complementation was reproduced with liquid media of the same supplementation (Fig 3B). These results demonstrate that AfuABC is sufficient to transport G6P and F6P as substrates for bacterial metabolism and growth. We did not observe complementation with *afuA* or *afuBC* alone, consistent with a model where AfuABC acts as a BPDT to transport substrates from the periplasm into the cytosol (Fig 3A).

### Conservation of AfuABC in bacteria

**Fig 4:**
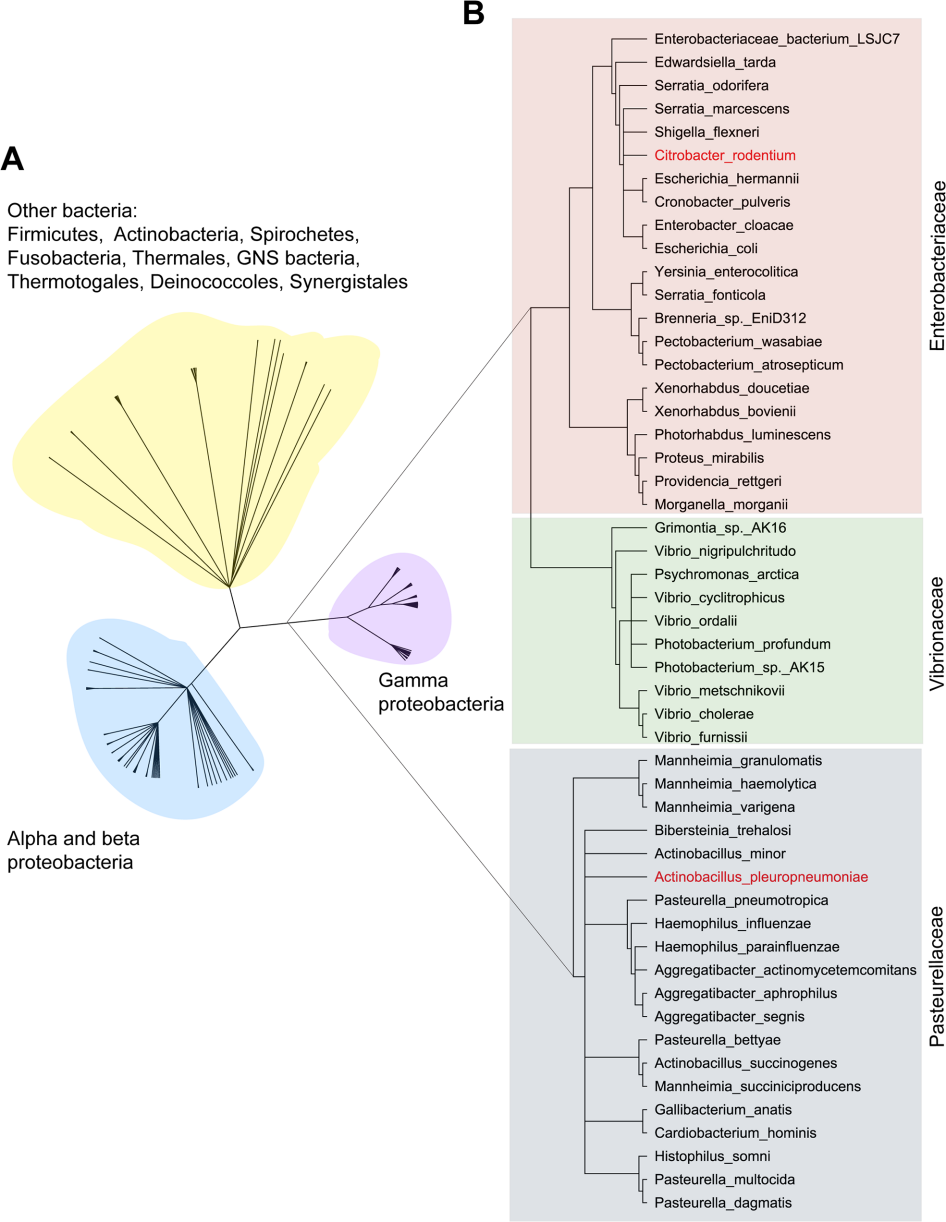
Phylogenetic tree of AfuA. (A) The complete tree of all 268 putative AfuA homologues. Based on the tree, homologues were divided into: gammaproteobacteria (83 hits, purple), alpha and betaproteobacteria (121 hits, cyan) and other bacteria (64 hits, yellow). (B) The phylogenetic tree of AfuA in gammaproteobacteria. The 83 gammaproteobacteria AfuA homologues were reduced to 51 by removing sequences that shared >95% identity. These hits were divided into: 21 in the *Enterobacteriaceae* (red), 10 present in the *Vibrionaceae* (green), and 20 in the *Pasteurellaceae* (grey). The two AfuA homologues used in this study are highlighted in red.

Given the novel function of AfuABC as a sugar-phosphate transporter, we next sought to identify whether this machinery was conserved in other bacteria. The three crystal structures provided a means to identify the sugar-coordinating residues (H205, D206 and E229) as the critical determinants in mediating binding of sugar-phosphates. These residues were subsequently used to define a potential sugar-phosphate binding motif, which in turn was used to search for and identify putative homologues of AfuA and hence the AfuABC operon (S5 Table and Fig 4). The majority of hits were found in Gram-negative bacteria. In particular, AfuABC was identified in many human pathogens from the *Pasteurellaceae* (e.g. *Haemophilus influenzae*), the *Vibrionaceae* (e.g. *Vibrio cholerae*) and the *Enterobacteriaceae* (e.g. *E*. *coli* O157:H7). Surprisingly, a minority of putative *afuABC* loci were also found in Gram-positive bacteria such as *Clostridium tetani* and other members of the Firmicutes. The conservation of AfuABC over a broad spectrum of bacterial genera suggests sugar-phosphate transport is required across a wide range of colonization niches and conditions.

### AfuABC contributes to the pathogenesis of *C*. *rodentium*

**Fig 5:**
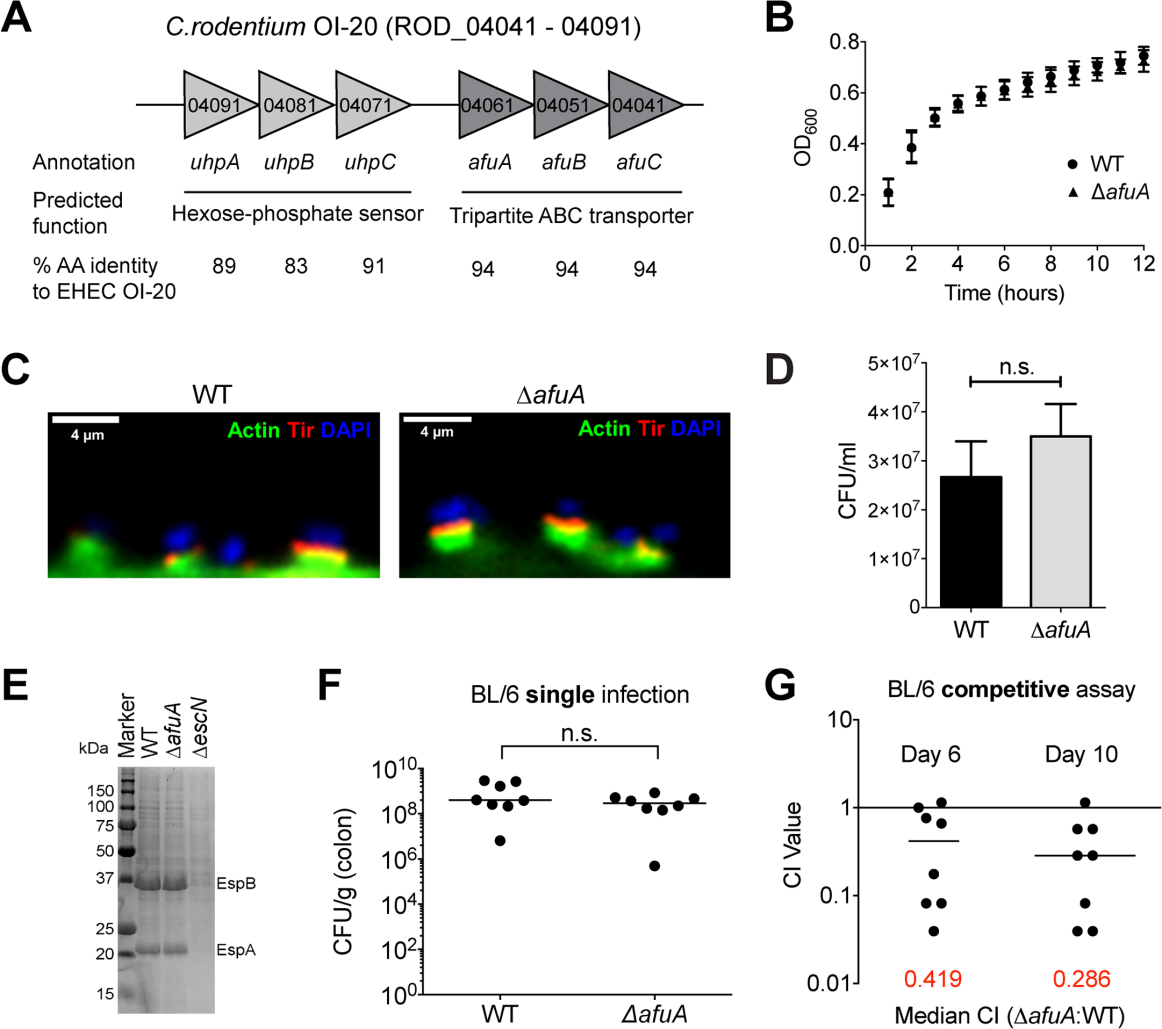
*C*. *rodentium* requires AfuA during host colonization. (A) Gene organization, annotation and predicted functions for genomic region corresponding to OI-20 in *C*. *rodentium*. Percent identity to EHEC O157:H7 was calculated with ClustalW2 using the entire predicted sequence of each protein. (B) Growth curves in LB broth for WT and *ΔafuA C*. *rodentium*. Cultures were tracked with OD_600_ readings for the indicated amount of time (n = 2). (C) Actin pedestal formation by *C*. *rodentium* WT or *ΔafuA*. HeLa cells were infected for 8hrs with *C*. *rodentium* then stained with phalloidin (green), anti-*C*. *rodentium* Tir (red) and DAPI to detect DNA (blue). (D) Adherence of *C*. *rodentium* WT and *ΔafuA* to HeLa cells after 8hrs infection. Counts shown represent adherent bacteria on a monolayer of cells (see Methods) and are mean values ± SEM (n = 3 infections). (E) SDS-PAGE of type-3 secretion effectors by *C*. *rodentium* WT, *ΔafuA* and *ΔescN* after growth in DMEM. *ΔescN* is a negative control strain that is secretion-deficient. (F) C57BL/6 mice were orally infected with *C*. *rodentium* WT or *ΔafuA* and bacterial colonic burdens determined at 10 dpi. Data were combined from two independent experiments. Each symbol represents one animal (n = 8 for each infection). The median is indicated. (G) Competitive index (CI) of simultaneous *C*. *rodentium* WT and *ΔafuA* infection in C57BL/6 mice in stool 6 dpi, and colonic tissue 10 dpi. Data were combined from two independent experiments (n = 8). A CI < 1 indicates the WT strain outcompeted the knockout (6 dpi median CI = 0.49, p = 0.0313; 10 dpi median CI = 0.38, p = 0.0156).

To examine whether AfuABC plays a role in bacterial pathogenesis, we utilized the intestinal murine pathogen *C*. *rodentium*, a commonly used model for EHEC O157:H7 as well as other attaching and effacing pathogens [15]. In EHEC, *afuABC* is located on the putative pathogenicity island OI-20. OI-20 also contains a recently identified fucose-sensing two-component regulatory system, suggesting this genomic island may play a role in carbohydrate sensing and/or acquisition [16]. OI-20 is conserved to >83% amino acid identity in *C*. *rodentium* ICC168 (Fig 5A), and ITC and growth assays confirmed that *C*. *rodentium* AfuABC functioned as a sugar-phosphate transporter [17,18] (S6 Table and S3 Fig). We generated an AfuA knockout strain of *C*. *rodentium* (*ΔafuA*) which did not display significant changes in planktonic growth, intestinal epithelial adherence/pedestal formation, type III effector secretion and bacterial tissue localization (Figs 5B–5E and S4).

Oral infection of C57BL/6 mice by *C*. *rodentium* WT or *ΔafuA* yielded no significant difference in bacterial colonic tissue burdens between the two groups at 10 days post infection (dpi) (Fig 5F). However, *C*. *rodentium* is a highly adapted mouse pathogen and strains lacking important virulence factors can still reach high colonic burdens in individual infections [19,20]. This prompted us to perform a competitive index (CI) assay to measure the comparative fitness between WT and *ΔafuA* in a simultaneous infection. *ΔafuA* was significantly impaired compared to WT in stool pellets at 6 dpi (median CI = 0.42, p = 0.031) as well as in colonic tissues at 10 dpi (CI = 0.29, p = 0.015) (Fig 5G) indicating that AfuABC is required for *C*. *rodentium* optimal colonization of the intestine. A competitive defect of this magnitude is comparable to those previously reported for *C*. *rodentium* strains lacking known secreted effectors [19–21].

### AfuABC substrates are present in the infected mouse gut

**Fig 6:**
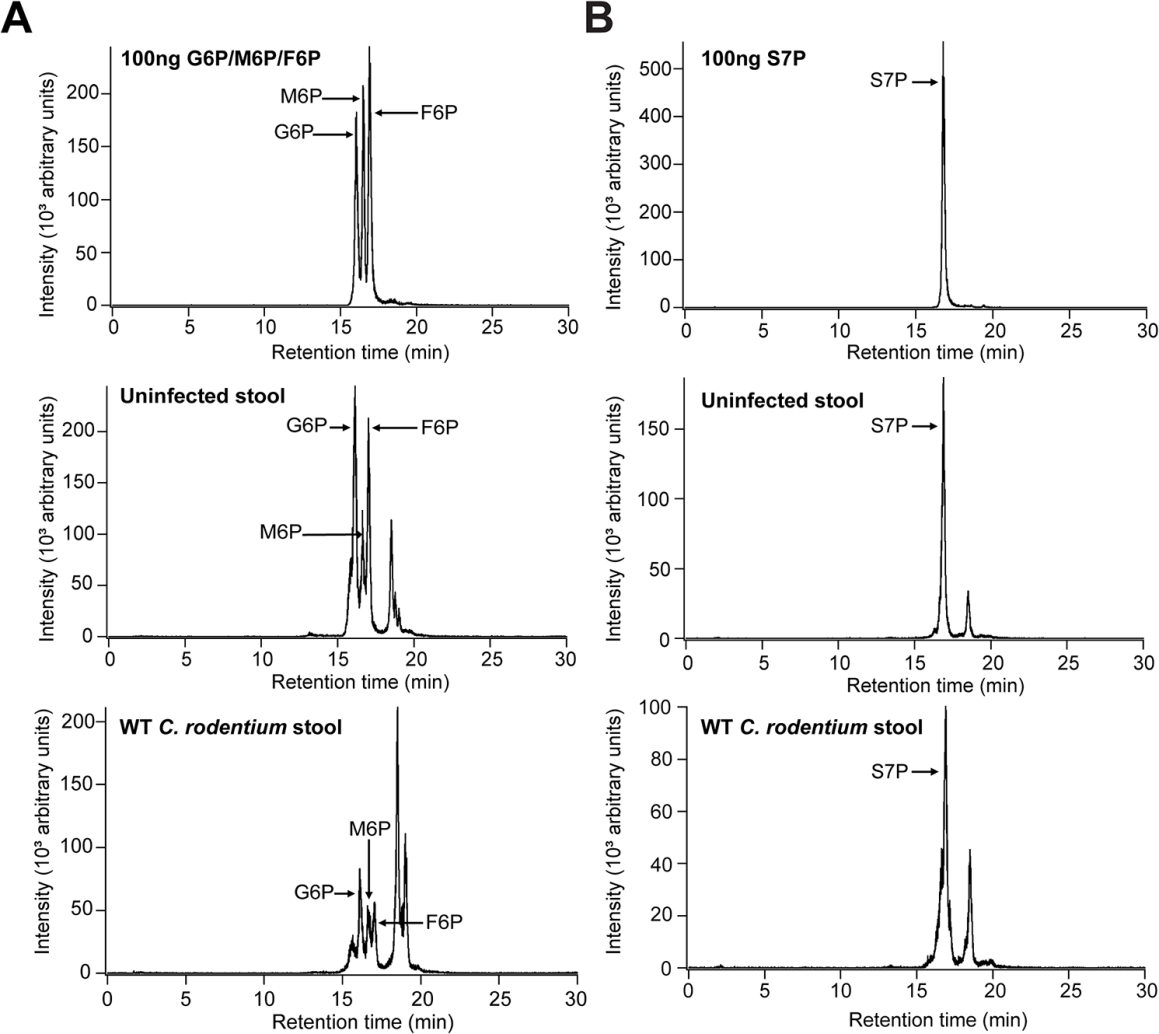
AfuABC substrates are present in the intestine during *C*. *rodentium* infection. (A) Representative LC-MS/MS chromatograms with indicated peaks for 100ng G6P/F6P/M6P mix (top), uninfected stool (middle) and *C*. *rodentium* WT infected stool (bottom). (B) S7P peaks. G6P/F6P/M6P were identified with a 259→97 ion transition and S7P was identified with a 289→97 ion transition.

The primary role of sugar-phosphates as cytosolic metabolites suggests that they would not be present in the extracellular environment and hence available for an extracellular pathogen such as *C*. *rodentium*. However, recent evidence has emerged indicating that they are present in the intestine and their availability is dependent on the presence of the host microbiota [22,23]. Since previous studies have not defined the concentration of these molecules, we used targeted liquid chromatography/tandem mass spectrometry to quantify the level of AfuABC substrates accessible to *C*. *rodentium* in control and *C*. *rodentium-*infected mice colons. Both validated substrates of AfuABC (G6P and F6P) as well as the other AfuA ligands (M6P and S7P) were present in all locations sampled (luminal contents, stool pellets and mucus scrapings) at micromolar quantities (Table 3 and Fig 6). These amounts exceed the K_d_ of *C*. *rodentium* AfuA for G6P or F6P, indicating that these sugar-phosphates are present in the mouse intestine at levels sufficient to be utilized by the bacteria.

**Table 3:** LC-MS/MS quantification of AfuA substrates across two sets of *C*. *rodentium*-infected mice.

	Set #1				Set #2			
	**Colon luminal contents (ng/extract)**							
	**G6P**	**F6P**	**M6P**	**S7P**	**G6P**	**F6P**	**M6P**	**S7P**
**UI**	446	276	122	29.5	163	150	49.6	23.2
**WT**	481	269	171	48.3	236	197	96.4	41.0
***ΔafuA***	210	109	45.7	20.5	23.5	13.5	6.5	3.09
	**Mucus scrapings (ng/extract)**							
	**G6P**	**F6P**	**M6P**	**S7P**	**G6P**	**F6P**	**M6P**	**S7P**
**UI**	85.2	57.4	27.2	6.64	496	347	189	17.2
**WT**	150	80.8	33.2	10.9	65.7	40.4	8.33	2.28
***ΔafuA***	54.5	26.6	10.5	4.36	94.4	21	12.2	n.d.
	**Fresh stool pellets (ng/extract)**							
	**G6P**	**F6P**	**M6P**	**S7P**	**G6P**	**F6P**	**M6P**	**S7P**
**UI**	364	493	165	168	575	330	76.1	117
**WT**	493	296	98	123	240	127	92.2	67.2
***ΔafuA***	1100	584	447	220	34.8	21.9	55.1	26.7

### AfuABC plays a critical role in transmission success of *C*. *rodentium*

**Fig 7:**
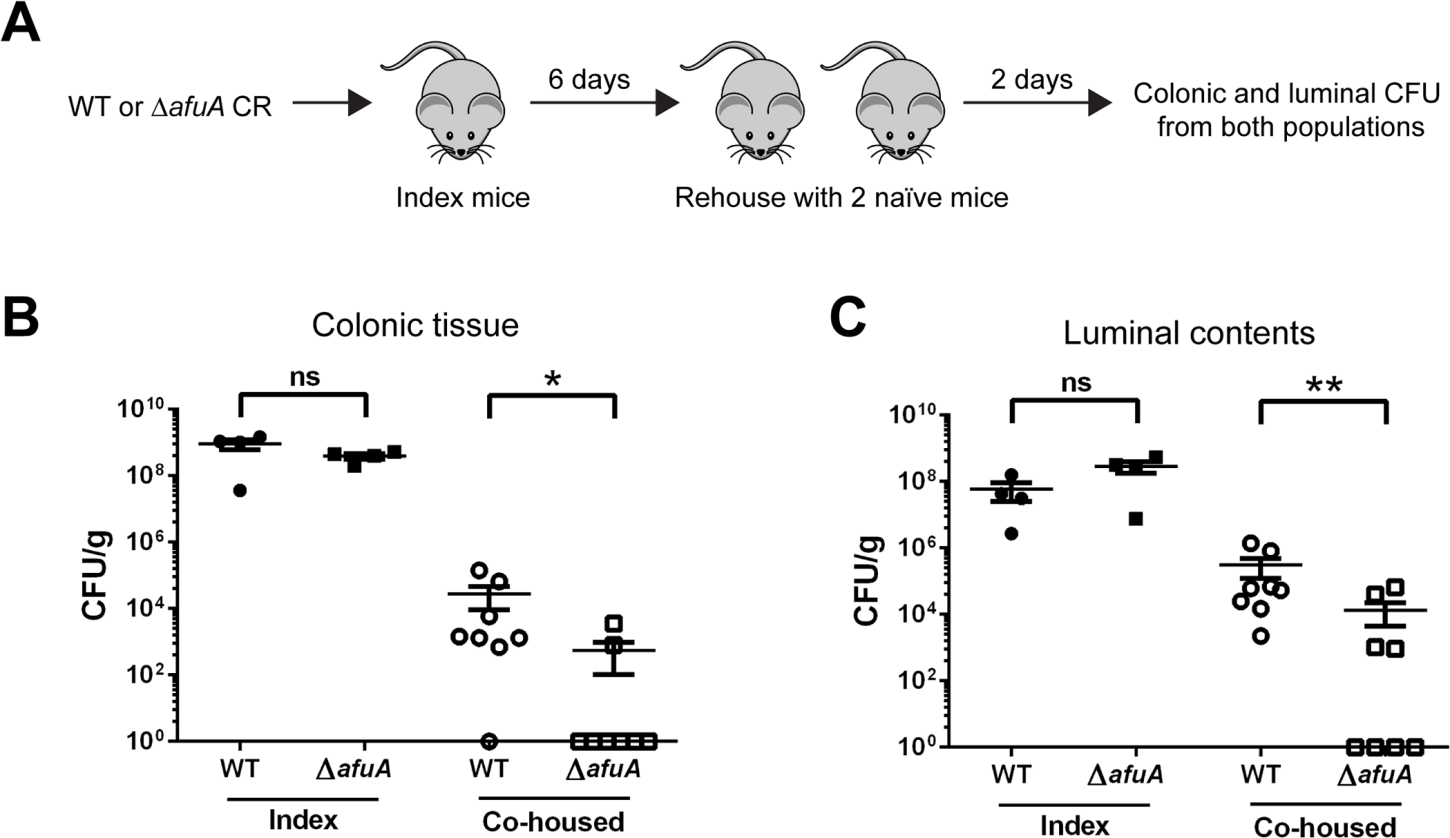
*C*. *rodentium* requires AfuA for efficient host-to-host transmission. (A) Infection schematic for quantification of *C*. *rodentium* host-to-host transmission. C57BL/6 index mice were orally infected with *C*. *rodentium* WT or *ΔafuA*. At 6 dpi, index mice were added to a cage containing two naïve mice and 48h post-exposure, bacterial luminal and colonic burdens determined. (B, C) Ability of *C*. *rodentium* WT or *ΔafuA* to transmit between index and co-housed C57BL/6 mice. Bacterial colonic (B) and luminal (C) burdens were determined for both index and co-housed mice. Data were combined from two independent experiments. Each symbol represents one animal (n = 4 (index), 8 (co-housed) for each infection). The median is indicated.* = p<0.05.

Typical assays with *C*. *rodentium* involve the oral delivery of large doses of bacteria to ensure reproducible infections. As such, they fail to mimic how *C*. *rodentium* and other enteric bacterial pathogens typically spread via the oral-fecal route in wild host populations. The high levels of sugar-phosphates present in shed stool pellets led us to examine a potential role for AfuA in the transmission of *C*. *rodentium* in a murine population. Index mice were orally infected with either WT or *ΔafuA* and after six days, co-housed with two naïve mice for 48 hours to allow transmission via coprophagy (Fig 7A). Seven out of eight co-housed mice exposed to WT-infected index mice were successfully infected, with heavy pathogen burdens recovered from both their colonic tissues and lumen (Fig 7B and 7C). In contrast, only half of the *ΔafuA* exposed mice showed any detectable *C*. *rodentium* within their luminal contents, and only two out of eight mice showed any *C*. *rodentium* adherent to their colonic tissues (Fig 7B and 7C). These results indicate that AfuABC does not directly affect the growth or infective capacity of *C*. *rodentium* but rather impacts its ability to compete and survive within the host intestinal environment and ultimately transmit to new hosts.

## Discussion

We conclude that AfuABC has been misannotated and is a sugar-phosphate transporter that contributes to the virulence of an enteric pathogen. We have defined a potential sugar-phosphate binding motif in AfuA and used it to identify a broad conservation of AfuABC across bacteria, including both Gram-negative and-positive species. Although AfuA was not required for *in vivo* colonization by *C*. *rodentium* or functional type III secretion, loss of AfuA significantly impacted its ability to be transmitted between hosts, indicating that sugar-phosphate uptake is required for a functional C. *rodentium* infective life cycle.

Competition between the host and bacteria for iron is an established concept in bacterial pathogenesis [24]. Therefore, a locus such as *afuABC* that had been misannotated as a Fe^3+^ transporter might have been assumed to contribute to virulence without direct experimental evidence indicating such a function. Previous data implicating AfuABC in iron uptake was based on the *afuABC-*mediated rescue of *ΔaroB* (siderophore-deficient) *E*. *coli* on iron-limited media [3]. However, since *aroB* encodes a metabolic enzyme that has dual roles in both carbon metabolism (the shikimate pathway) and siderophore synthesis, it is not clear whether the *afuABC* complementation of *ΔaroB* observed was only attributable to a complementation of iron uptake in this strain. Since the sole function of UhpT is in hexose-phosphate uptake, the assay we used in this study is more specific and directly implicates AfuABC in transport of these substrates instead of iron. Based on the results presented here, AfuABC does contribute to bacterial pathogenesis but this is achieved through sugar-phosphate and not iron uptake. It remains an open question whether other predicted iron transporters may, akin to AfuABC, be misannotated.

The structures shown here are the first structural data for a non-enzymatic sugar-phosphate (i.e. G6P) binding protein. In AfuA, the essential residues His205 and Asp206 form multivalent hydrogen bonds to the sugar moiety of ligands and function as the primary determinants of ligand selectivity. On the opposing end of the pocket, redundant phosphate-coordinating residues such as Ser37 cooperate to hold the phosphate moiety of ligands. This bipartite binding cleft is able to discriminate between isomers of the same sugar-phosphate (G6P vs. G1P) and between differing orientations of side chains around the carbohydrate ring (F6P vs. R5P). Our observation that both furan (F6P and S7P) and pyran (G6P) ligands could bind AfuA suggests the position of hydroxyl determinants is more important than steric fitting of the overall ring structure into the binding pocket.

We propose a two-step mechanism for ligand capture by AfuA. Substrates such as G6P or F6P first dock to the protein via hydrogen bonding to residues in the electronegative region of the binding cleft. Next, the negatively charged phosphate group of the ligand serves as the target of a simultaneous rotation and closure of the two globular domains of AfuA, generating a stable AfuA-ligand complex. This cinching mechanism is driven by the electrostatic attraction of the Arg/Lys-rich regions of the binding pocket (which also includes residues such as Ser37 and Thr150). This mechanism is compatible with our mutagenesis data, as all mutations to sugar-coordinating residues completely eliminated binding, suggesting initial recognition of the sugar ring is the determining step in ligand capture. Other homologues of AfuA may display altered substrate specificity and thus might provide more insight into the mechanism of sugar recognition and coordination by PBPs.

We have confirmed AfuABC functions as a novel sugar-phosphate uptake mechanism. The only other known transporter in Gram-negative bacteria for sugar-phosphates is UhpABCT [4]. The primary distinction between these two systems is that AfuABC is an active transport system that relies on cytosolic hydrolysis of ATP, whereas UhpT-mediated transport is dependent on the maintenance of a phosphate/sugar-phosphate concentration gradient. Since AfuABC does not rely on a concentration gradient nor the concomitant phosphate efflux which is required by UhpT, uptake of sugar-phosphates through AfuABC may be preferable in a location where nutrients are constantly in flux (such as the intestinal lumen and mucosa). AfuABC-mediated transport could potentially allow a pathogen to simultaneously conserve phosphate stores and take advantage of nutrients for which other bacteria may lack an appropriate transport mechanism [4]. It is established that sugar-phosphate uptake is important for intracellular pathogen replication [25–27]. Our study provides evidence that the same is true for extracellular pathogens and provides reference values for these ligands in the mouse intestine. While the most likely downstream fate of transported sugar-phosphates through AfuABC is to support bacterial metabolism, recent studies have identified both phosphorylated and cognate sugars as molecules that can act as signaling intermediaries in the host-bacterial interaction [16,28]. The close genetic proximity of AfuABC to one of these systems in both *C*. *rodentium* and EHEC O157:H7, the FusKR system, implies that the substrates targeted by AfuABC could also be involved in other roles besides pure carbon and phosphate metabolism.

The identification of AfuABC homologues in bacteria that colonize a variety of niches, such as the gastrointestinal and respiratory tracts, suggests uptake of sugar-phosphates plays a role in their competitive establishment on these surfaces. Since the microbiota is known to modulate the nutritional environment of these areas, we propose that AfuABC could aid in pathogen competition with host-associated commensal microbes during colonization. Notably, the clear dependence of successful pathogen transmission on the presence of AfuABC supports this concept since our assays were performed at early time points, when *C*. *rodentium* would need to overcome colonization resistance by the resident commensal microbes in order to successfully infect the host [29]. The major contributing source of sugar-phosphates to the gut metabolome remains unclear. It is likely that a variety of host and microbial factors contribute to the pool of these substrates (e.g. from epithelial cell turnover and bacterial lysis). One potential source of sugar phosphates is the mucin family of glycosylated cell surface proteins. The contributions of mucin-derived substrates, in particular fucose and sialic acid, to bacterial infections have recently been recognized in several studies [16,30–32]. Intriguingly, when EHEC is grown in the presence of mucus, the OI-20 pathogenicity island is upregulated, indicating a potential connection between AfuABC and the presence of mucin [33]. Mice lacking the predominant intestinal mucin Muc2 mice are hypersusceptible to *C*. *rodentium* infection due to decreased mucin-dependent bacterial clearance and it will be interesting to determine if this and other mucins are involved in generation of free sugar-phosphates in the gut [34]. Further characterization of the source and flux of concentration of sugar-phosphates during dysbiosis will help to illuminate the potential role of the microbiota in generating substrates for AfuABC and the contribution of sugar-phosphate transport within mammalian hosts.

## Materials and Methods

### Bacterial strains and growth

All bacteria were grown at 37°C on Luria-Bertani (LB) agar plates or in LB broth with shaking. A list of strains and plasmids used in this study is included in S7 Table. Where appropriate, antibiotics were supplemented at the following concentrations: kanamycin – 50μg/mL, ampicillin – 50μg/mL, streptomycin—100μg/mL.

### Cloning and expression of AfuA and AfuABC

AfuA was initially cloned into a pET26b expression vector from *A*.*pleuropneumoniae* genomic DNA. For the growth assays, complementation vectors with low (PSC101) copy numbers were used. *afuA* or *afuABC* were amplified from *A*. *pleuropneumoniae* genomic DNA and cloned into each backbone by restriction free or exponential megaprimer cloning, respectively [35,36]. Plasmid insert sequences were confirmed by forward and reverse capillary sequencing (The Centre for Applied Genomics, Toronto, Canada).

### Purification of AfuA

*E*. *coli* transformed with an AfuA expression plasmid were grown overnight in LB-kanamycin and used to initiate liter-scale volumes of the same media. Population density was estimated by OD_600_ and IPTG was added to a concentration of 1mM when the OD_600_ reached 0.6. Cells were induced at 20°C for 16 hours, after which they were spun down at 4000rpm at 4°C. Pellets were resuspended in a 20% sucrose solution to initiate osmotic shock and spun down again at 4000rpm. Sucrose pellets were resuspended in a 5mM MgCl_2_ lysis solution with added protease inhibitors (Roche Applied Sciences, Canada) and allowed to incubate on ice for at least 20 minutes. The fluid was then centrifuged at 30,000rpm for 40 minutes at 4°C in an ultracentrifuge (Beckman Coulter, USA). The supernatant was passed through a 0.45μM syringe filter and batch bound for >1 hour to Ni-NTA resin (Pierce, USA) at 4°C with shaking. The solution was then passed through a gravity column and washed with wash buffer (20mM imidazole, 50mM Tris pH 8 and 300mM NaCl). A 6M solution of guanidine hydrochloride was used to denature bound protein after which a 3M Gd-HCl solution and finally pure wash buffer was applied to allow bound protein to refold. Protein was eluted with elution buffer (wash buffer with 400mM imidazole) and purity assessed with denaturing SDS-PAGE followed by Coomassie blue staining. Fractions with detectable AfuA were pooled and dialyzed with added thrombin (Sigma-Aldrich, USA) in wash buffer to simultaneously remove excess imidazole and cleave the N-terminal 6xHis tag from AfuA. The solution was spun down and concentrated in a 10kDa cutoff centrifugal filter (Millipore, USA). Samples were further purified on a Superdex S75 10/300 gel filtration column (GE Healthcare, USA) pre-equilibrated with a buffer consisting of 20mM Tris (pH 8) and 100mM NaCl. Fraction purity was assessed by SDS-PAGE with Coomassie Blue staining. Since we initially investigated AfuA in the context of iron capture, initial protein purifications in this study leading to the structure of G6P-AfuA were performed in the absence of guanidine. All other purifications used for ITC and crystallography were denaturing preparations.

### Site-directed mutagenesis

To generate single-residue mutants of AfuA, site-directed mutagenesis was performed on the original pET26b-APAfuA vector. A short (17 cycle) PCR reaction was used to generate the desired mutation in the template plasmid. PCR products were digested with DpnI at 37°C for 4–6 hours to cleave undesired template plasmid and were then used to transform competent *E*. *coli* (NEB Turbo) for selection. Mutations were confirmed by forward and reverse capillary sequencing (The Centre for Applied Genomics, Toronto, Canada).

### Isothermal titration calorimetry (ITC)

ITC was performed using a VP-ITC instrument (Microcal, GE Healthcare). Runs consisted of ligand loaded into the injection syringe titrated against purified protein loaded into the sample cell. All solutions were pre-dialyzed in buffer for at least 12 hours to mitigate effects of dilution. The buffer used was the same as the gel filtration buffer described above and power changes were measured relative to a reference cell filled with distilled water. Runs consisted of a 10:1 ligand:protein ratio, with efforts made to adhere to a 200μM:20μM concentration standard. Data used to calculate binding constants were referenced against runs performed with ligand alone to control for the heat of ligand solvation.

### Crystallization, data collection and structure solution

For crystallization trials, AfuA was purified as described above and concentrated to at least 10mg/mL. A common crystallization condition consisting of 0.2M MgCl_2_, 0.1M Tris pH 6.5 and 25% PEG3350 was found for AfuA bound to either G6P, F6P or S7P. Apo-AfuA crystallized best in 0.2M MgCl_2_, 0.1M MES:NaOH pH 6.0 and 27.5% PEG 3350. Candidate crystals were looped in a cryoprotectant solution consisting of the mother liquor with 20% glycerol and flash frozen in liquid N_2_. The sulfur anomalous diffraction data for AfuA bound to G6P was collected at the Structural Genomics Consortium (SGC) (Toronto, Canada) using a chromium rotating anode X-ray source (λ= 2.29 Å) and R-AXIS IV^++^ detector (Rigaku). To improve the signal-to-noise ratio of the anomalous signal of sulfur, the crystal was scanned over 326° and a dataset was collected with an average I/σI of 59 with 11 fold redundancy to 2.3 Å resolution. Data was collected for AfuA-G6P, AfuA-F6P and apo-AfuA at beamline 08ID-1 (CMCF-ID) at the Canadian Light Source (CLS) (Saskatchewan, Canada). For AfuA-S7P, data was collected at NECAT beamline 24-ID-E at the Advanced Photon Source (APS) (Chicago, USA). Sulfur single wavelength anomalous dispersion (SAD) phasing was used to phase the initial G6P-bound structure of AfuA. This structure was then used as a molecular replacement (MR) search model for the solutions of AfuA-F6P and AfuA-S7P. For apo-AfuA, the AfuA-G6P structure was split into two domains (correlating to the two lobes of the protein), each of which was used as an independent search model for MR. All MR and automated refinement was performed with the PHENIX suite [37]. Manual refinement was undertaken with Coot [38]. Chimera and PyMol were used for molecular modeling, graphics (PyMol) and electrostatic surface mapping (Chimera) [39]. Atomic coordinate files were deposited in the Protein Data Bank under the accession numbers 4R72 (Apo), 4R73 (G6P), 4R74 (F6P) and 4R75 (S7P).

### *E*. *coli* complementation assays

For growth assays, both the mutant (*ΔuhpT*) and the WT strain (BW25113) were obtained from the Keio collection of single gene knockouts [40]. Overnight cultures were grown in 5mL of M9 minimal media + 10mM glucose + antibiotics. Cells were washed twice in 1mL M9 salts (no carbon) and brought to a density of 10^9^ cells/mL. For solid agar growth assays, a dilution series of 5μL spots from 10^7^ to 10^3^ cells/mL was used. Plates were incubated at 37°C for 30 hours and were imaged with a GelDoc XR system (BioRad). For liquid growth assays, cells were plated at dilutions ranging from 10^8^ to 10^4^ cells/mL in wells containing 100μL of the appropriate minimal media. Each well was duplicated to account for pipetting errors. Plates were incubated at 37°C with shaking in a Bioscreen C microplate reader (Growth Curves USA). Growth was measured by OD_600_ readings every 15 minutes for at least 24 hours. For data analysis, cell density of duplicate wells was averaged. All assays were performed in at least triplicate.

### Bioinfomatics and phylogenetic tree construction

*A*. *pleuropneumoniae* AfuA was used as a template for a BlastP search. The results were filtered by removing homologs that did not possess the substrate specificity-determining residues. To reduce the tree size further, AfuA hits from multiple strains of the same organism were removed, keeping the top hit with highest sequence similarity. The selected hits used for further analysis came from 268 different organisms. The multiple sequence alignments and phylogenetic tree (neighbor-joining) construction was performed with Geneious R7 (Biomatters). MUSCLE [41] was used to generate the multiple sequence alignment. The tree was re-sampled 100 times using the bootstrap module and braches with less than 60% confidence were trimmed. The tree was confirmed by using the MrBayes [42] module.

### *C*. *rodentium* growth curve

Strains from an overnight culture were diluted 1/100 into fresh LB, and 200 μl transferred into a sterile 96-well plate in triplicate (Costar). The optical density at 600 nm was measured every 15 min after 5s of orbital shaking using a Trilux Scintillation Counter (Wallec).

### Generation of *afuA* mutant *C*.*rodentium*

DBS100 in-frame deletion mutants were generated using the suicide vector pRE118 via *sacB*-based allelic exchange [43]. A 1.5kb fragment upstream of *afuA* was amplified by primers (GT*GGTACC*TGCGCGAGCGCGTCAGCGCG and CC*GCTAGC*CGCCGCCAGCGCTACGGCAGAG) with flanking *Kpn*I and *Nhe*I sites, while a 1.5kb fragment downstream of *afuA* was amplified by primers (CC*GAGCTC*TGCTGCCGTAGAGCAGGGCG and CC*GCTAGC*TACGGCTCAACGGAGGTGC) with flanking *Sac*I and *Nhe*I sites (italicised text indicates restriction sites). Upstream and downstream fragments were digested alongside pRE118 with appropriate restriction enzyme and cloned into *E*. *coli* SY327 λpir to generate deletion vector pΔ*afuA*. DBS100 was electroporated with pΔ*afuA* and grown on kanamycin. Resulting colonies were inoculated on sucrose plates and a third set of primers (GTCGCTGGTTATTGAACGC and GGATGCAGAGCGAGTGTCTG) was used to confirm deletion of the gene for sucrose resistant, kanamycin sensitive colonies.

### Mouse infections and competitive index (CI) assays

C57BL/6 mice (8–12 weeks old) were bred under specific pathogen-free conditions at the Child and Family Research Institute. *C*. *rodentium* WT (DBS100) and *ΔafuA* strains were grown shaking overnight at 37°C in 3 ml of LB. Cell density was measured by OD_600_ readings. Mice were orally gavaged with 100 μl of either WT, *ΔafuA* or a 1:1 mix of WT to Δ*afuA* (total 2.5 × 10^8^ cfu). Stool was collected at day 6 pi, homogenized, serial diluted and plated on LB agar supplemented with 100 μg/ml of streptomycin. Animals were euthanized at 10 dpi and colonic tissues were removed, homogenized and plated. For CI, single colonies were picked and used as templates for colony PCR with deletion screening primers from Δ*afuA* generation. CI was calculated as the ratio of *ΔafuA* to WT colonies divided by the ratio of *ΔafuA* to WT from the input. All mouse experiments were performed in accordance with protocols approved by the University of British Columbia’s Animal Care Committee and in direct accordance with the Canadian Council on Animal Care’s guidelines.

### Mouse transmission experiments

Assessment of the ability of *C*. *rodentium* to transmit between hosts was adapted from the protocol used by Wickham *et al* [44]. Index C57BL/6 mice were infected with WT or Δ*afuA C*. *rodentium* as outlined above. At 6 dpi, the index mouse plus two naïve mice (referred to as “co-housed”) were added to a new cage. Naïve mice remained co-housed with the index mouse for 48 h, at which time they were euthanized. Their colon tissues and luminal contents were then homogenized, serial diluted and plated on LB agar supplemented with 100 μg/ml of streptomycin for enumeration of *C*. *rodentium* burdens.

### Profiling T3SS effectors in WT and *ΔafuA C*. *rodentium*

As previously described [45,46], *C*. *rodentium* WT, *ΔafuA* and *ΔescN* were streaked onto LB-streptomycin plates for single colony isolation. 5 ml LB-streptomycin was inoculated with a single colony of above mentioned strains and grown shaking O/N at 37°C. The strains were then subcultured at a 1:50 dilution into Dulbecco's modified Eagle's medium (DMEM). The bacteria were incubated statically at 37°C, 5% CO_2_ until the optical density of the cultures reached 0.7 (OD_600_ ~0.7). Bacteria were pelleted (13200 rpm, 4°C, 10 minutes) and proteins in the supernatant were precipitated using 10% trichloroacetic acid (TCA, Sigma) O/N at 4°C. Precipitated proteins were pelleted by centrifugation (13200 rpm, 4°C, 10 minutes) and resuspended in Laemmli buffer. Samples were resolved on 12% SDS-polyacrylamide gels and visualized by Coomassie R-250 Blue staining.

### *In vitro C*. *rodentium* adherence and pedestal formation

HeLa cells were seeded at a concentration of 5x10^4^ cells per well and incubated for 24 h prior to infection. To initialize bacterial infection, tissue cultures were pre-incubated in 1 ml of DMEM (Life Technologies) supplemented with 2% FBS for 30 min. Cells were then infected with an overnight culture of *C*. *rodentium* DBS100 at a MOI of 1:100 for 8 hours. Monolayers were washed three times with Dulbecco's PBS (Life Technologies) to remove nonadherent bacteria, and treated with 200μl of 0.1% Triton X-100 PBS for 5 min at room temperature. Samples were serially diluted and incubated on LB-streptomycin plates overnight at 37°C. Values are the mean CFU of three independent experiments repeated in triplicate, and statistical comparisons between groups were performed with two-tailed Student’s *t* tests. Sterile round coverslips were also seeded and infected as above. After infection, coverslips were washed three times with Dulbecco's PBS and fixed in 4% paraformaldehyde (Fisher Scientific) overnight. The fixed cells were washed three times in PBS and permeabilized by incubation in 0.1% Triton X-100 in PBS for 10 min. Cover slips were incubated in anti-Tir rat polyclonal IgG antibody diluted 1:2000 in 1% BSA in TBST for 1 hour. The coverslips were then washed three times in TBST and incubated in Alexa Fluor 568 goat anti-rat IgG (diluted 1:1000) and phalloidin-Alexa Fluor 488 conjugate (Pierce) in 1% BSA in TBST for 1 hour. After three washes in PBS, cells were mounted with ProLong Gold Mountant with DAPI (Life Technologies). Images were acquired on a Zeiss AxioImager Z1 with an AxioCam HRm camera operating through AxioVision software.

### Fluorescence microscopy of *C*. *rodentium-*infected colonic tissue

Immunofluorescence staining of infected tissues was performed using previously described procedures [47]. In brief, colon tissues were fixed in 10% neutral buffered formalin (Fisher) for 16 hrs, rinsed in 70% ethanol and paraffin embedded. Sectioning was completed by the histology laboratory at the Child and Family Research Institute. Serial 5 μm sections were cut and deparaffinized by heating at 60°C, xylene treatment and rehydration through an ethanol gradient to water. Sections were treated with PBS containing 0.1% Triton X-100, followed by blocking buffer (PBS containing 0.05% Tween 20 and 1% normal donkey serum). Tissues were incubated with goat anti-cytokeratin 19 (1:300, Santa Cruz Biotechnology) and rabbit anti-*C*. *rodentium* Tir (1:5,000; gift from W. Deng) and subsequently probed with Alexa Fluor 568-conjugated donkey anti-goat IgG (1:1000; Life Technologies) and Alexa Fluor 488-conjugate donkey anti-rabbit IgG (1:1000; Life Technologies). Tissues were mounted and imaged in the same manner as the *in vitro* pedestal coverslips.

### Quantification of sugar-phosphates in murine intestinal tissues

C57BL/6 mice were infected with *C*. *rodentium* WT or *ΔafuA* as described above for single-strain infections. At 6 dpi, shed stool pellets were collected and mice sacrificed. From each individual, colons were extracted, cut longitudinally, and luminal contents removed. Mucus was collected via gentle scraping along the apical colonic surface with a glass slide. Samples were immediately resuspended in 25mM HEPES pH 7.0 at a ratio of 1μL:1mg tissue, gently shaken to dissolve soluble extracellular species and stored on ice. Suspensions were centrifuged and equal volumes of supernatant from each sample were subjected to a conventional MeOH/CHCl_3_ extraction. Aqueous layers were kept and dried in a vacuum centrifuge and stored at -80°C for further analysis. Samples were analyzed with targeted tandem liquid chromatography/mass spectrometry (LC-MS/MS) at the Analytical Facility for Bioactive Molecules (Hospital for Sick Children, Canada) using an optimized protocol for LC separation of sugar-phosphates. Samples were separated on a Synergi Hydro-RP column (Phenomenex) and quantified with an API 4000 triple quadrupole mass spectrometer (Sciex). Quantification was performed against a standard curve subjected to the extraction process.

### Statistical analysis

The mean values ± standard errors of the means (SEM) for at least two independent experiments is shown in all figures unless stated otherwise. p values were calculated using a Wilcoxon Rank Sum Test (CI) or a nonparametric Mann-Whitney t-test using GraphPad Prism software (v6.05). A p value less than 0.05 was considered statistically significant.

## Supporting Information

S1 Fig Representative ITC outputs for *A*. *pleuropneumoniae* AfuA titrated with potential ligands. (A-I) ITC curves obtained for AfuA titrated wth (A): F6P, (B) S7P, (C) M6P, (D) G1P, (E) R5P, (F) F1,6BP, (G) fructose, (H) mannose, (I) Na_2_HPO_4_. A binding event is clearly identifiable in A-C as a sigmoidal shaped curve. All others may be classified as non-binding. The minor slope exhibited in Panel F most likely derives from contaminating F6P in the ligand stock. Each curve shown is representative of protein from at least 3 independent AfuA purifications. * denotes the anomeric carbon in M6P in Panel C.
(TIF)

S2 Fig Additional data for rescue of Δ*uhpT* by *afuABC* complementation. (A) OD_600_ readings of *ΔuhpT E*.*coli* complemented with *uhpT* over 24 hours at 37°C in M9 minimal medium supplemented with 10mM glucose, G6P, fructose or F6P. Readings were taken every 15 minutes–data shown is parsed to hourly readings for clarity. Curve legend is the same in all panels and is indicated in the first panel. Error bars represent SEM of cell growth from n = 3 transformations. (B) Rescue on M9 MM + 10mM F6P agar plates. Details of the experiment are identical to that of Fig 3A in the main manuscript. The plates shown are representative of n = 3 independent transformations.
(TIF)

S3 Fig Characterization of AfuABC from *C*.*rodentium*. (A) ITC curves obtained for *C*. *rodentium* AfuA titrated with G6P (left) or glucose (right). (B) Complementation of Δ*uhpT* by *C*.*rodentium afuABC* in liquid M9 MM supplemented with either 10mM glucose, fructose, G6P or F6P. Values shown are the mean OD_600_ ± SEM of cultures grown for 24 hours at 37°C from n = 3 transformations. * = p<0.05, ** = p<0.005 by an unpaired t-test.
(TIF)

S4 Fig Localization of *C*. *rodentium* WT and *ΔafuA* in the mouse colon. C57BL/6 mice were orally infected with *C*. *rodentium* WT or *ΔafuA*. Colon tissues (10 dpi) were stained with anti-cytokeratin 19 (red), anti-*C*. *rodentium* Tir (green) and DAPI to detect DNA (blue). Images were acquired at 200x magnification.
(TIF)

S1 Table Data collection and refinement statistics for structures reported by this study. (DOCX)

S2 Table Thermodynamic values of WT *A*. *pleuropneumoniae* AfuA ligand binding. (DOCX)

S3 Table Mutagenesis ITC results for fructose-6-phosphate. (DOCX)

S4 Table Doubling times of AfuABC-complemented *ΔuhpT E*. *coli* strains. (DOCX)

S5 Table List of bacterial species harbouring AfuA homologues with a predicted sugar-phosphate binding motif. (XLSX)

S6 Table ITC-derived binding constants for *C*. *rodentium* AfuA. (DOCX)

S7 Table List of plasmids and strains used in this study. (DOCX)
