# Supplementary figures and images for: Active Transport of Phosphorylated Carbohydrates Promotes Intestinal Colonization and Transmission of a Bacterial Pathogen
Source: PLoS Pathog. 2015 Aug 21;11(8):e1005107. doi: 10.1371/journal.ppat.1005107 (PMC4546632; doi:10.1371/journal.ppat.1005107)

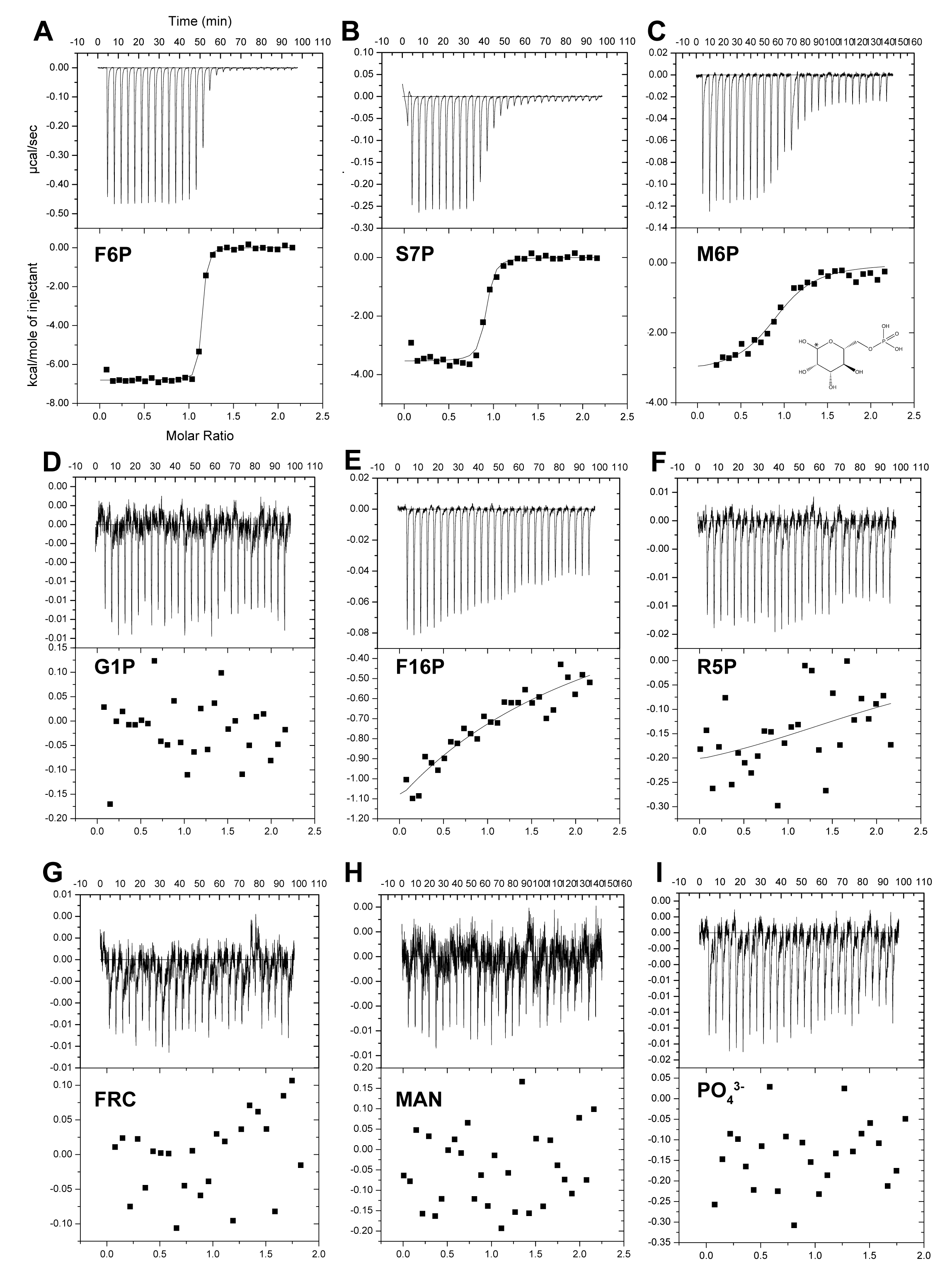

Supplement: S1 Fig — (A-I) ITC curves obtained for AfuA titrated wth (A): F6P, (B) S7P, (C) M6P, (D) G1P, (E) R5P, (F) F1,6BP, (G) fructose, (H) mannose, (I) Na2HPO4. A binding event is clearly identifiable in A-C as a sigmoidal shaped curve. All others may be classified as non-binding. The minor slope exhibited in Panel F most likely derives from contaminating F6P in the ligand stock. Each curve shown is representative of protein from at least 3 independent AfuA purifications. * denotes the anomeric carbon in M6P in Panel C. (TIF) [file ppat.1005107.s001.tif]

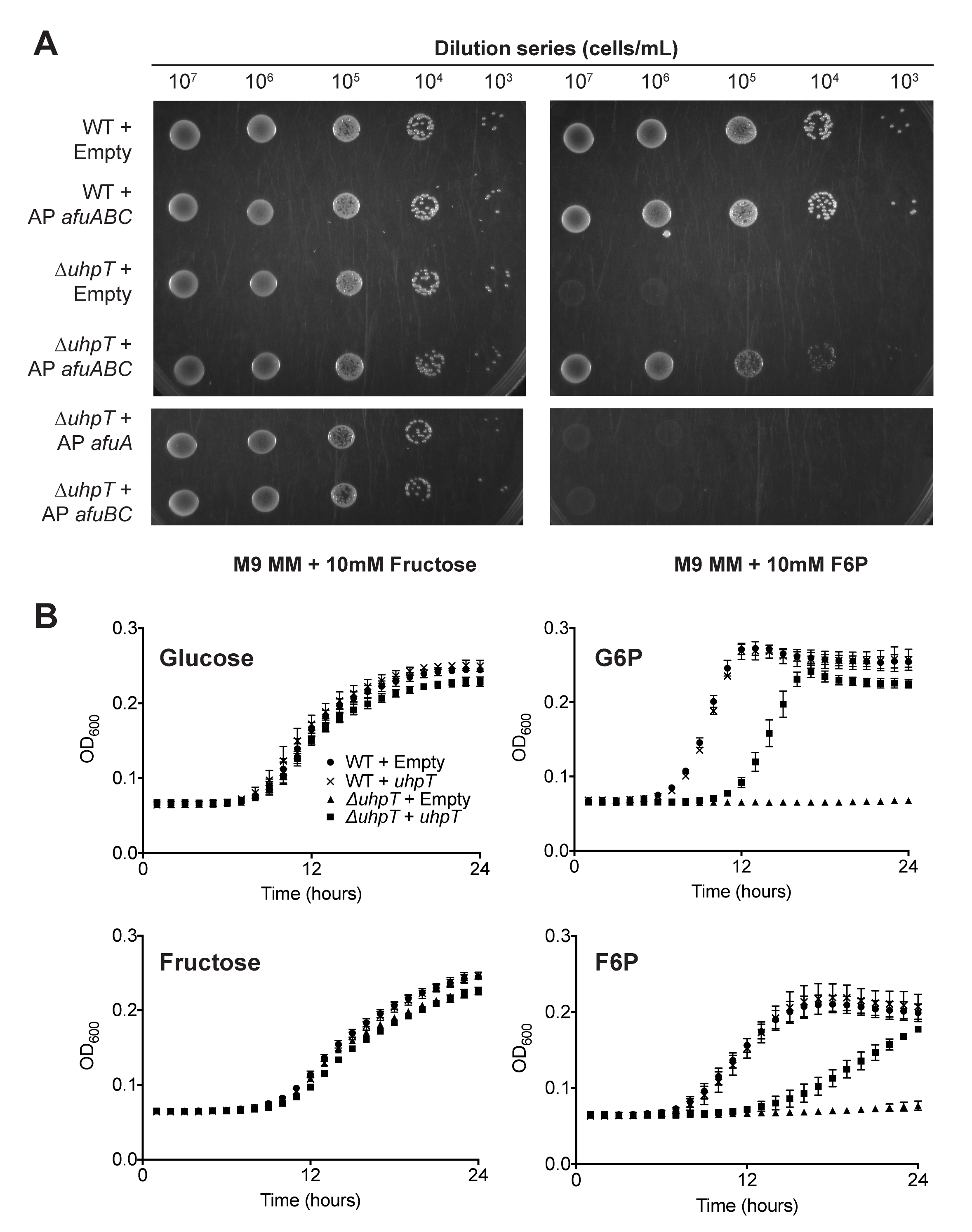

Supplement: S2 Fig — (A) OD600 readings of ΔuhpT E.coli complemented with uhpT over 24 hours at 37°C in M9 minimal medium supplemented with 10mM glucose, G6P, fructose or F6P. Readings were taken every 15 minutes–data shown is parsed to hourly readings for clarity. Curve legend is the same in all panels and is indicated in the first panel. Error bars represent SEM of cell growth from n = 3 transformations. (B) Rescue on M9 MM + 10mM F6P agar plates. Details of the experiment are identical to that of Fig 3A in the main manuscript. The plates shown are representative of n = 3 independent transformations. (TIF) [file ppat.1005107.s002.tif]

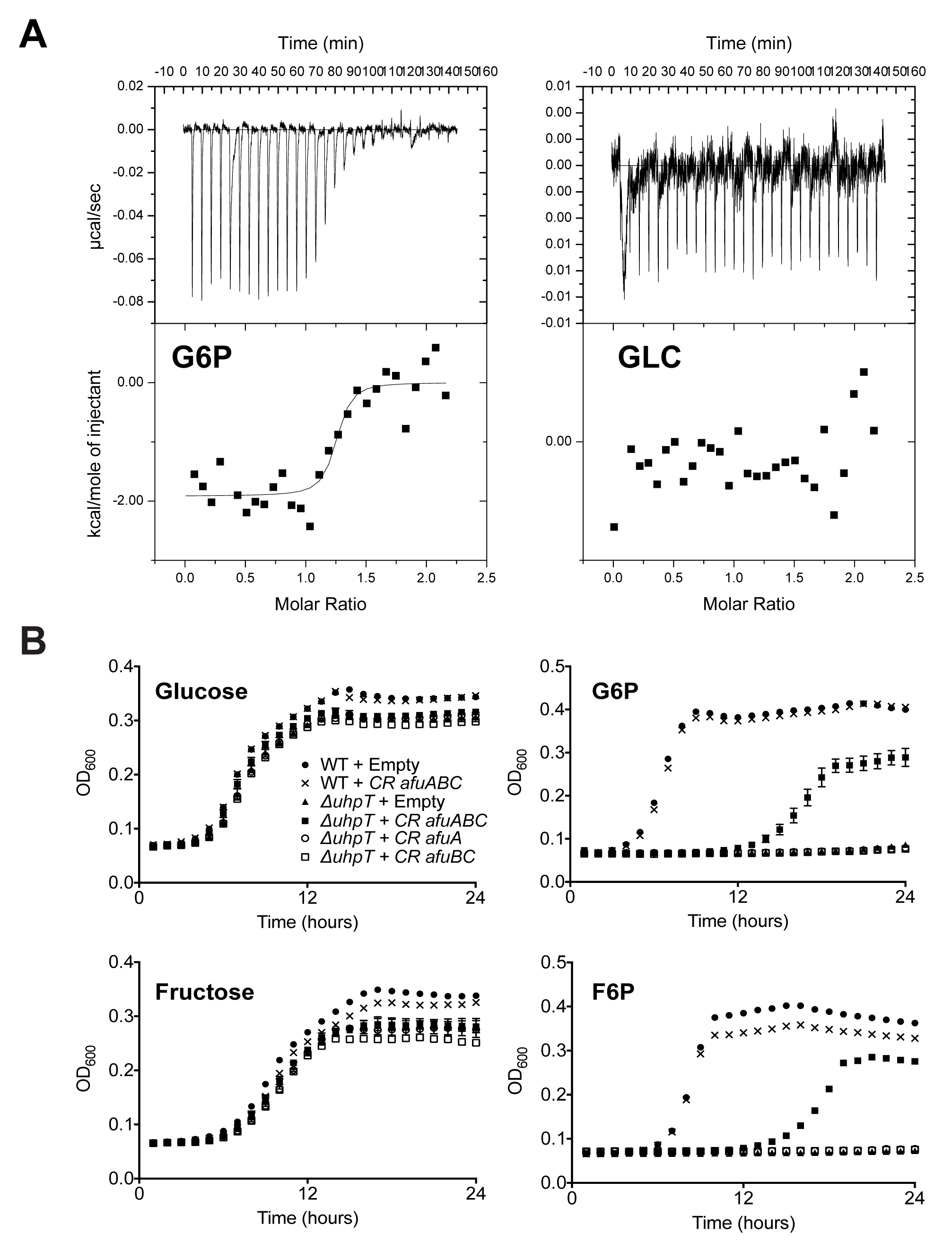

Supplement: S3 Fig — (A) ITC curves obtained for C. rodentium AfuA titrated with G6P (left) or glucose (right). (B) Complementation of ΔuhpT by C.rodentium afuABC in liquid M9 MM supplemented with either 10mM glucose, fructose, G6P or F6P. Values shown are the mean OD600 ± SEM of cultures grown for 24 hours at 37°C from n = 3 transformations. * = p<0.05, ** = p<0.005 by an unpaired t-test. (TIF) [file ppat.1005107.s003.tif]

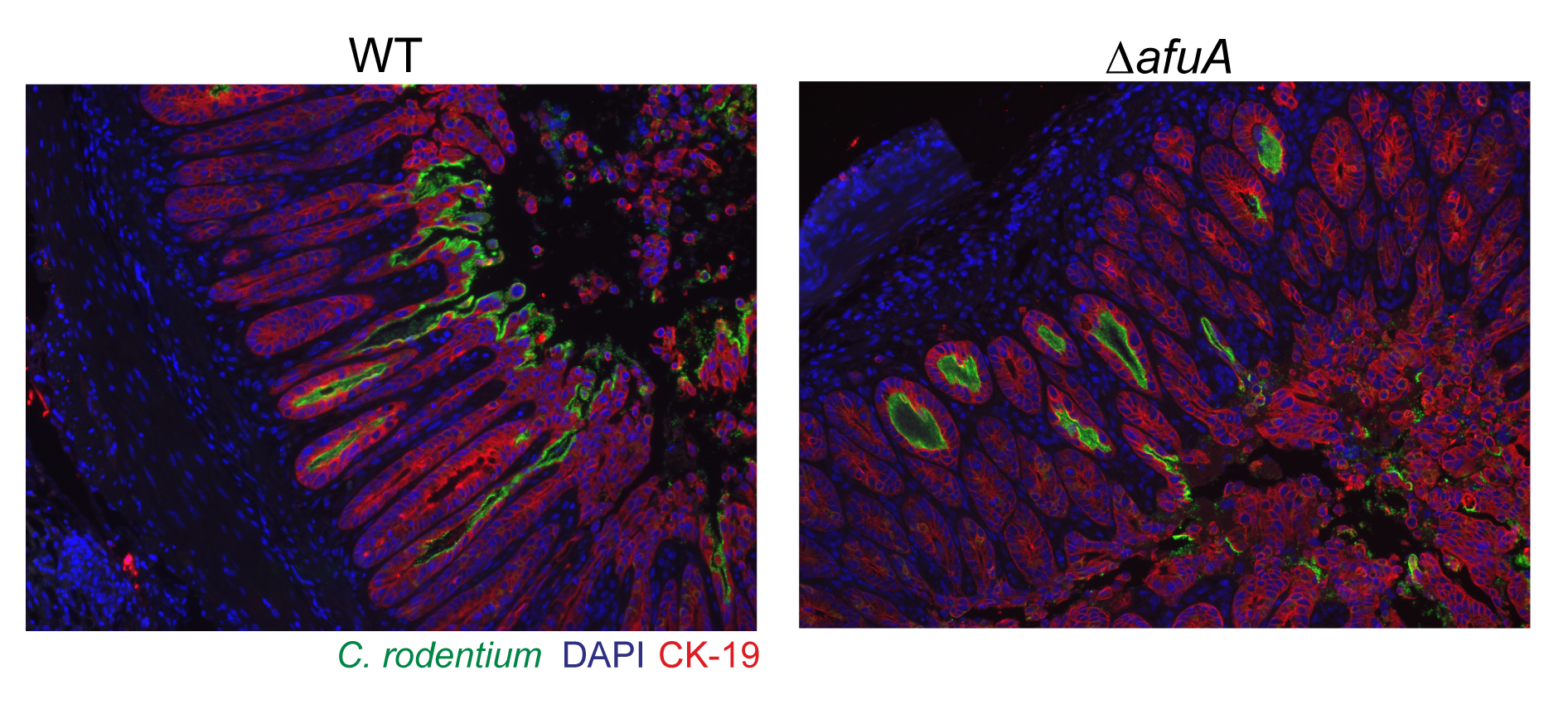

Supplement: S4 Fig — C57BL/6 mice were orally infected with C. rodentium WT or ΔafuA. Colon tissues (10 dpi) were stained with anti-cytokeratin 19 (red), anti-C. rodentium Tir (green) and DAPI to detect DNA (blue). Images were acquired at 200x magnification. (TIF) [file ppat.1005107.s004.tif]
