# Supplementary material for: Active Transport of Phosphorylated Carbohydrates Promotes Intestinal Colonization and Transmission of a Bacterial Pathogen
Source: PLoS Pathog. 2015 Aug 21;11(8):e1005107. doi: 10.1371/journal.ppat.1005107 (PMC4546632; doi:10.1371/journal.ppat.1005107)
Supplement: S1 Table — (DOCX) [file ppat.1005107.s005.docx]

**Table S1. Data collection and refinement statistics for structures reported by this study.**

|  | **AfuA-G6P** | **AfuA-F6P** | **AfuA-S7P** | **Apo-AfuA** |
| --- | --- | --- | --- | --- |
| **Data collection** |  |  |  |  |
| Space group | P 21 21 21 | P 1 21 1 | P 21 21 21 | C 2 2 21 |
| Cell dimensions (*a, b, c*) (Å) | 43.50, 95.30, 152.10 | 43.20, 96.99, 145.07 | 47.40, 59.90, 106.01 | 57.95, 80.82, 126.04 |
| Cell dimensions (α, β, γ) (°) | 90, 90, 90 | 90, 90, 90 | 90, 90, 90 | 90, 90, 90 |
| Resolution (Å) | 39.60-1.49 (1.7-1.6) | 48.49-1.93 (2.03-1.93) | 43.28-1.28 (1.35-1.28) | 47.09-1.6 (1.7-1.6) |
| R_merge_ | 7.7 (79) | 13.1 (81.7) | 6.1 (53.0) | 5.1 (85.6) |
| I/σI | 21.8 (2.6) | 14.2 (2.4) | 14.3 (2.0) | 20.8 (1.25) |
| Completeness (%) | 90.6 (81.8) | 100.0 (99.9) | 98.3 (97.5) | 95.4 (70.7) |
| Redundancy | 11.1 (10.5) | 7.6 (7.5) | 4.1 (3.8) | 6.2 (4.5) |
|  |  |  |  |  |
| **Refinement** |  |  |  |  |
| Resolution (Å) | 1.6 | 1.93 | 1.28 | 1.6 |
| No. unique reflections | 94866 | 89452 | 77210 | 44112 |
| R_work_/R_free_ | 0.19/0.21 | 0.15/0.20 | 0.20/0.23 | 0.18/0.22 |
| No. atoms (protein) | 4954 | 9987 | 2595 | 2551 |
| No. atoms (ligand/ion) | 54 | 264 | 36 | 24 |
| No. atoms (water) | 672 | 959 | 447 | 448 |
| β-factor (protein) | 17.34 | 19.98 | 13.32 | 20.61 |
| β-factor (ligand/ion) | 12.85 | 25.30 | 19.41 | 28.77 |
| β-factor (water) | 28.95 | 28.95 | 27.62 | 31.34 |
| Bond length RMSD (Å) | 0.006 | 0.0072 | 0.006 | 0.006 |
| Bond angle RMSD (°) | 1.119 | 1.068 | 1.062 | 1.068 |
|  |  |  |  |  |
| **PDB Accession Code** | 4R72 | 4R73 | 4R74 | 4R75 |

Values in parentheses are indicative of the highest resolution shell.

All datasets were obtained from single crystals.
