# Supplementary material for: Active Transport of Phosphorylated Carbohydrates Promotes Intestinal Colonization and Transmission of a Bacterial Pathogen
Source: PLoS Pathog. 2015 Aug 21;11(8):e1005107. doi: 10.1371/journal.ppat.1005107 (PMC4546632; doi:10.1371/journal.ppat.1005107)
Supplement: S2 Table — (DOCX) [file ppat.1005107.s006.docx]

**Table S2. Thermodynamic values of WT *A. pleuropneumoniae* AfuA ligand binding.**

| **Ligand** | **Binding Sites (N)** | **ΔH (kcal·mol^-1^)** | **ΔS (cal·mol^-1^·deg^-1^)** |
| --- | --- | --- | --- |
| **Glucose-6-phosphate** | 1.06 ± 0.04 | -5.00 ± 0.09 | 18.3 ± 0.4 |
| **Fructose-6-phosphate** | 1.18 ± 0.03 | -6.61 ± 0.09 | 14.9 ± 0.5 |
| **Sedoheptulose-7-phosphate** | 0.93 ± 0.08 | -3.70 ± 0.20 | 21.1 ± 1.8 |
| **Mannose-6-phosphate** | 0.98 ± 0.02 | -3.01 ± 0.17 | 17.5 ± 0.6 |
