# Supplementary material for: Active Transport of Phosphorylated Carbohydrates Promotes Intestinal Colonization and Transmission of a Bacterial Pathogen
Source: PLoS Pathog. 2015 Aug 21;11(8):e1005107. doi: 10.1371/journal.ppat.1005107 (PMC4546632; doi:10.1371/journal.ppat.1005107)
Supplement: S3 Table — (DOCX) [file ppat.1005107.s007.docx]

**Table S3. Mutagenesis ITC results for fructose-6-phosphate.**

| **Mutant** | **F6P K_d_ (µM)** | **F6P Sites (N)** |
| --- | --- | --- |
| **WT** | 0.008 ± 0.001 | 1.18 ± 0.03 |
| **S37A** | 1.3 ± 0.6 | 1.09 ± 0.01 |
| **S37D** | 3.3 ± 0.4 | 1.07 ± 0.02 |
| **T150A** | 2.1 ± 0.3 | 1.10 ± 0.03 |
| **H205A** | No binding | |
| **D206A** | No binding | |
| **E229A** | No binding | |
