# Supplementary material for: Active Transport of Phosphorylated Carbohydrates Promotes Intestinal Colonization and Transmission of a Bacterial Pathogen
Source: PLoS Pathog. 2015 Aug 21;11(8):e1005107. doi: 10.1371/journal.ppat.1005107 (PMC4546632; doi:10.1371/journal.ppat.1005107)
Supplement: S4 Table — (DOCX) [file ppat.1005107.s008.docx]

**Table S4. Doubling times of AfuABC-complemented *ΔuhpT* *E. coli* strains.**

| **Strain** | **Doubling time (minutes)** | | | |
| --- | --- | --- | --- | --- |
|  | **Glucose** | **G6P** | **Fructose** | **F6P** |
| **WT + empty** | 228 | 104 | 203 | 107 |
| **WT + AP *afuABC*** | 206 | 105 | 189 | 114 |
| ***ΔuhpT* + empty** | 175 | n.g. | 217 | n.g. |
| ***ΔuhpT* + AP *afuABC*** | 167 | 194 | 250 | 249 |

Doubling times were calculated from the linear portion of the mean growth curves in Figure 3.

n.g. = no growth detected
