# Supplementary material for: Active Transport of Phosphorylated Carbohydrates Promotes Intestinal Colonization and Transmission of a Bacterial Pathogen
Source: PLoS Pathog. 2015 Aug 21;11(8):e1005107. doi: 10.1371/journal.ppat.1005107 (PMC4546632; doi:10.1371/journal.ppat.1005107)
Supplement: S6 Table — (DOCX) [file ppat.1005107.s010.docx]

**Table S6. ITC-derived binding constants for *C. rodentium* AfuA.**

| **Ligand** | **K_d_ (nM)** | **Binding Sites (N)** | **ΔH (kcal·mol^-1^)** | **ΔS (cal·mol^-1^·deg^-1^)** |
| --- | --- | --- | --- | --- |
| **Glucose-6-phosphate** | 164 ± 94 | 1.18 ± 0.03 | -3.35 ± 1.50 | 20.7 ± 6.0 |
| **Fructose-6-phosphate** | 326 | 1.28 | -5.5 | 11.0 |
| **Glucose** | No binding | | | |
| **Fructose** | No binding | | | |
| **Phosphate** | No binding | | | |
