# Supplementary material for: Active Transport of Phosphorylated Carbohydrates Promotes Intestinal Colonization and Transmission of a Bacterial Pathogen
Source: PLoS Pathog. 2015 Aug 21;11(8):e1005107. doi: 10.1371/journal.ppat.1005107 (PMC4546632; doi:10.1371/journal.ppat.1005107)
Supplement: S7 Table — (DOCX) [file ppat.1005107.s011.docx]

**Table S7. Strains and plasmids used in this study.**

| **Strain or plasmid** | **Description** | **Reference** |
| --- | --- | --- |
| **Strains** |  |  |
| *Citrobacter rodentium* DBS100 | Strep^R^ | [1] |
| *Citrobacter rodentium* Δ*afuA* | DBS100 ΔafuA | This study |
| *Escherichia coli* BL21 (DE3) | *fhuA*2 [lon] *ompT* gal (λ DE3) [dcm] ∆*hsdS*; λDE3 = λ sBamHIo ∆EcoRI-B int::(*lacI*::p*lacUV*5::T7 gene1) i21 ∆nin5 | Novagen |
| *Escherichia coli* MM294 | F-, *glnV*44(AS) *rfbC*1 *endA*1 *spoT*1 thi-1 *hsdR*17 *creC*510 | [2] |
| *Escherichia coli* BW25113 | F-, DE(*araD-araB*)567, *lacZ*4787(del)::*rrnB*-3, LAM-, rph-1, DE(*rhaD-rhaB*)568, *hsdR*514 | [3] |
| *Escherichia coli* JW3641-2 | BW25113 Δ*uhpT* | [3] |
| *Escherichia coli* SY327 λpir | *th*-1*thr leu tonA lacY supE*, *recA*::RP4-2-Tc::Mu (*λpir*) | [4] |
|  |  |  |
| **Plasmids** |  |  |
| pRE118 | *oriT oriV sacB* (Suc^S^) *ahpA* (Km^R^) | [5] |
| pΔ*afuA* | Deletion construct for *afuA* in *C. rodentium*; Kan^R^ Suc^S^ | This study |
| pET26b-HisAfuA | Expression vector for *A. pleuropneumoniae* WT NHis-AfuA; Kan^R^ | This study |
| pET26b-HisAfuA-S37A | Expression vector for *A. pleuropneumoniae* S73A NHis-AfuA; Kan^R^ | This study |
| pET26b-HisAfua-S37D | Expression vector for *A. pleuropneumoniae* S37D NHis-AfuA; Kan^R^ | This study |
| pET26b-HisAfuA-T150A | Expression vector for *A. pleuropneumoniae* T150A NHis-AfuA; Kan^R^ | This study |
| pET26b-HisAfuA-H205A | Expression vector for *A. pleuropneumoniae* H205A NHis-AfuA; Kan^R^ | This study |
| pET26b-HisAfuA-D206A | Expression vector for *A. pleuropneumoniae* D206A NHis-AfuA; Kan^R^ | This study |
| pET26b-HisAfuA-E229A | Expression vector for *A. pleuropneumoniae* E229A NHis-AfuA; Kan^R^ | This study |
| pET26b-CRHisAfuA | Expression vector for *C. rodentium* NHis-AfuA; Kan^R^ | This study |
| pSC101-empty | Custom vector with pSC101 ori; Amp^R^ | A. Schryvers |
| pSC101-ECUhpT | pSC101 vector + *E. coli* UhpT with -262bp sequence of APAfuABC; Amp^R^ | This study |
| pSC101-APAfuABC | pSC101 vector + *A. pleuropneumoniae* AfuABC with -262bp upstream; Amp^R^ | This study |
| pSC101-APAfuA | pSC101 vector + *A. pleuropneumoniae* AfuA with -262bp upstream; Amp^R^ | This study |
| pSC101-APAfuBC | pSC101 vector + *A. pleuropneumoniae* AfuBC with -262bp upstream; Amp^R^ | This study |
| pSC101-CRAfuABC | pSC101 vector + *C. rodentium* AfuABC with -262bp sequence of APAfuABC; Amp^R^ | This study |
| pSC101-CRAfuA | pSC101 vector + *C. rodentium* AfuA with -262bp sequence of APAfuABC; Amp^R^ | This study |
| pSC101-CRAfuBC | pSC101 vector + *C. rodentium* AfuBC with -262bp sequence of APAfuABC; Amp^R^ | This study |

**Supporting Information References:**

1. Schauer DB, Falkow S (1993) Attaching and effacing locus of a Citrobacter freundii biotype that causes transmissible murine colonic hyperplasia. Infect Immun 61: 2486–2492.

2. Meselson M, Yuan R (1968) DNA restriction enzyme from E. coli. Nature 217: 1110–1114.

3. Baba T, Ara T, Hasegawa M, Takai Y, Okumura Y, et al. (2006) Construction of Escherichia coli K-12 in-frame, single-gene knockout mutants: the Keio collection. Mol Syst Biol 2: 2006.0008. doi:10.1038/msb4100050.

4. Miller VL, Mekalanos JJ (1988) A novel suicide vector and its use in construction of insertion mutations: osmoregulation of outer membrane proteins and virulence determinants in Vibrio cholerae requires toxR. J Bacteriol 170: 2575–2583.

5. Edwards RA, Keller LH, Schifferli DM (1998) Improved allelic exchange vectors and their use to analyze 987P fimbria gene expression. Gene 207: 149–157. doi:10.1016/S0378-1119(97)00619-7.
